# Catalytic Function of PLA2G6 Is Impaired by Mutations Associated with Infantile Neuroaxonal Dystrophy but Not Dystonia-Parkinsonism

**DOI:** 10.1371/journal.pone.0012897

**Published:** 2010-09-23

**Authors:** Laura A. Engel, Zheng Jing, Daniel E. O'Brien, Mengyang Sun, Paul T. Kotzbauer

**Affiliations:** Departments of Neurology and Developmental Biology, Hope Center for Neurological Disorders, Washington University School of Medicine, St. Louis, Missouri, United States of America; National Institutes of Health, United States of America

## Abstract

**Background:**

Mutations in the *PLA2G6* gene have been identified in autosomal recessive neurodegenerative diseases classified as infantile neuroaxonal dystrophy (INAD), neurodegeneration with brain iron accumulation (NBIA), and dystonia-parkinsonism. These clinical syndromes display two significantly different disease phenotypes. NBIA and INAD are very similar, involving widespread neurodegeneration that begins within the first 1–2 years of life. In contrast, patients with dystonia-parkinsonism present with a parkinsonian movement disorder beginning at 15 to 30 years of age. The *PLA2G6* gene encodes the PLA2G6 enzyme, also known as group VIA calcium-independent phospholipase A_2_, which has previously been shown to hydrolyze the sn-2 acyl chain of phospholipids, generating free fatty acids and lysophospholipids.

**Methodology/Principal Findings:**

We produced purified recombinant wildtype (WT) and mutant human PLA2G6 proteins and examined their catalytic function using *in vitro* assays with radiolabeled lipid substrates. We find that human PLA2G6 enzyme hydrolyzes both phospholipids and lysophospholipids, releasing free fatty acids. Mutations associated with different disease phenotypes have different effects on catalytic activity. Mutations associated with INAD/NBIA cause loss of enzyme activity, with mutant proteins exhibiting less than 20% of the specific activity of WT protein in both lysophospholipase and phospholipase assays. In contrast, mutations associated with dystonia-parkinsonism do not impair catalytic activity, and two mutations produce a significant increase in specific activity for phospholipid but not lysophospholipid substrates.

**Conclusions/Significance:**

These results indicate that different alterations in PLA2G6 function produce the different disease phenotypes of NBIA/INAD and dystonia-parkinsonism. INAD/NBIA is caused by loss of the ability of PLA2G6 to catalyze fatty acid release from phospholipids, which predicts accumulation of PLA2G6 phospholipid substrates and provides a mechanistic explanation for the accumulation of membranes in neuroaxonal spheroids previously observed in histopathological studies of INAD/NBIA. In contrast, dystonia-parkinsonism mutations do not appear to directly impair catalytic function, but may modify substrate preferences or regulatory mechanisms for PLA2G6.

## Introduction

Mutations in the *PLA2G6* gene (Entrez GeneID:8398) have been identified in autosomal recessive neurodegenerative diseases classified as infantile neuroaxonal dystrophy (INAD), neurodegeneration with brain iron accumulation (NBIA), and dystonia-parkinsonism [1]–[3]. Although there is significant overlap between the NBIA and INAD phenotypic spectrum, the clinical features of dystonia-parkinsonism are distinct in many ways from those reported for NBIA and INAD. INAD and NBIA caused by *PLA2G6* mutations typically begin in the first two years of life and involve progressive impairment of movement, speech and cognition, secondary to widespread degeneration in the peripheral and central nervous system [4]–[6]. Additional clinical features specific for the INAD/NBIA phenotypic spectrum include cerebellar atrophy and iron accumulation in the globus pallidus, both of which can be observed on magnetic resonance imaging of the brain. In contrast, dystonia-parkinsonism begins primarily as a movement disorder in the age range of 15–30 years old, and is further distinguished from NBIA/INAD by the absence of cerebellar atrophy and iron accumulation [2], [3]. A combination of dystonia and parkinsonism are the common presenting features, and similar to idiopathic PD, the parkinsonism is responsive to levodopa or a dopamine receptor agonist. Cognitive impairment is observed with disease progression.

The *PLA2G6* gene encodes group VIA calcium-independent phospholipase A2 (PLA2G6) also known as calcium-independent phospholipase A2 beta (iPLA_2_β). The enzyme was originally identified in Chinese hamster ovary cells based on its ability to hydrolyze the sn-2 acyl groups of phospholipids, producing free fatty acids and lysophospholipids [7], [8]. Morgan et al originally mapped a gene locus containing *PLA2G6* in multiple families with autosomal recessive inheritance of INAD or NBIA [1]. Sequencing of the *PLA2G6* gene in INAD and NBIA revealed a total of 44 unique mutations associated with disease. In all but one case in which *PLA2G6* mutations were detected, mutations were present in both alleles, indicating that disease is caused by loss of function rather than a dominant gain of function. In some INAD/NBIA cases, both alleles were affected by early frame shift and stop codon mutations, suggesting a complete loss of protein function [1], [6]. However the majority of disease-associated mutations cause missense single amino acid substitutions.

Subsequent studies identified *PLA2G6* mutations in patients with dystonia-parkinsonism. Paisan-Ruiz et al identified regions of homozygosity on chromosome 22 in two families with dystonia-parkinsonism [2]. Sequencing of genes in this region revealed missense mutations in *PLA2G6*, causing amino acid substitutions R741Q in one family and R747W in the other. In each case, affected patients were homozygous for the missense mutation in *PLA2G6*. A third missense mutation in *PLA2G6*, causing amino acid substitution R632W has been identified in association with dystonia-parkinsonism in 3 siblings [3]. The three affected siblings in this family were homozygous for the missense mutation, while 3 unaffected siblings and parents were heterozygotes. Interestingly, the R632W mutation has been identified on one allele in an INAD patient with compound heterozygous mutations in *PLA2G6* [1].

Distinct phenotypes associated with mutations in the same gene may result from the influence of additional genetic and environmental factors. Alternatively, individual *PLA2G6* mutations may primarily determine phenotype through distinct effects on protein function, causing either different degrees of impairment in a single function, or perhaps affecting different functions of the same protein. To examine the hypothesis that disparate phenotypes are determined primarily by distinct effects of mutations on PLA2G6 enzyme function, we developed assays to assess the catalytic activity of wildtype (WT) and mutant PLA2G6 proteins. We find that the human PLA2G6 enzyme functions as an A2 phospholipase, hydrolyzing the sn-2 acyl chain of phosphatidylcholine (PC), and as a lysophospholipase, hydrolyzing the sn-1 acyl chain of lysophosphatidylcholine (LPC), the product of its A2 phospholipase reaction. We find that mutations associated with INAD and NBIA profoundly impair enzyme function in both phospholipase and lysophospholipase assays. In contrast, mutations associated with dystonia-parkinsonism mutations do not impair catalytic function.

## Results

### Recombinant human PLA2G6 catalyzes the release of free fatty acids from multiple lipid substrates

**Figure 1:**
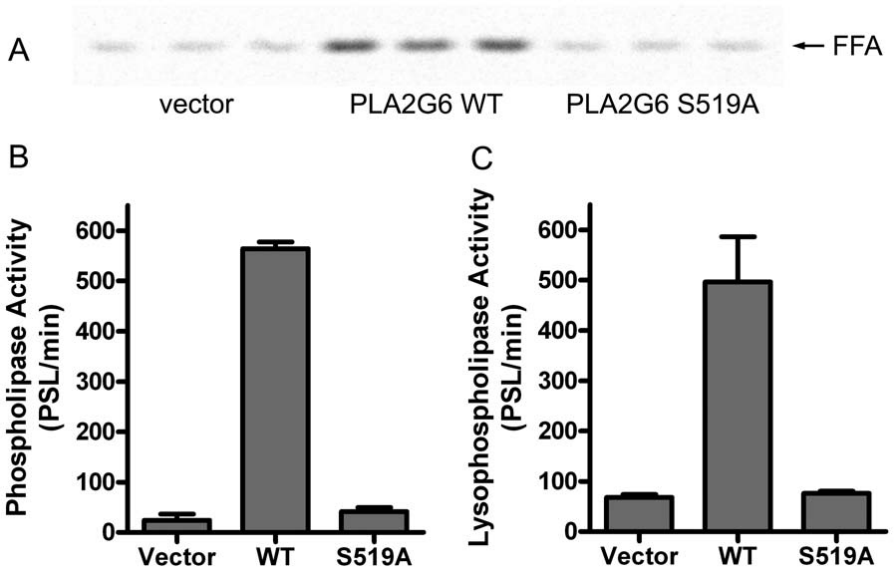
Recombinant human PLA2G6 protein catalyzes the hydrolysis of fatty acids from PC and LPC. (A) Purified recombinant protein preparations from cells transfected with an empty expression vector, WT PLA2G6 or S519A PLA2G6 were added to in vitro catalytic assays. Free fatty acids (FFA) released from ^14^C-labeled 1-palmitoyl lysophosphatidylcholine were separated on TLC and detected using a phosphorimager. Incubation of substrate with WT PLA2G6 enzyme produces robust release of fatty acids compared to control preparations from vector-transfected cells. Catalytic activity is abolished by mutation of S519 in the lipase catalytic site. (B) Quantitation of catalytic activity for the ^14^C-labeled phospholipid substrate 1-palmitoyl-2-oleyl phosphatidylcholine. (C) Quantitation of activity for ^14^C-labeled LPC lysophospholipid substrate. Fatty acid release in catalytic assays was quantitated from the phosphorimager screen in photostimulated luminescence (PSL) units, and the graphs indicate the rate of hydrolysis for each substrate, measured by the increase in PSL units per min of incubation time in each assay. Since the PLA2G6 protein concentrations, radiochemical specific activities and substrate concentrations were the same in both assays, the results indicate that the catalytic rates for the two substrates are similar.

We produced purified recombinant wildtype human PLA2G6 protein and used in vitro assays with radiolabeled lipid substrates to examine its catalytic function. Recombinant protein was produced by transient transfection of the 293FT cell line and purified using nickel affinity chromatography to capture a six histidine tag added to the C-terminus of PLA2G6. We produced recombinant protein for the longest PLA2G6 isoform, encoded by transcript variant 1, and examined its catalytic lipase activity using two lipid substrates (Figure 1). Recombinant PLA2G6 catalyzed the release of oleic acid from the phospholipid substrate 1-palmitoyl-2-oleyl-phosphatidylcholine (PC). Recombinant PLA2G6 also catalyzed the release of palmitic acid from the 2-lysophospholipid, 1-palmitoyl lysophosphatidylcholine (LPC). Lysophospholipids can be generated from phospholipids by the A2 phospholipase activity of PLA2G6 as well as other A2 phospholipase enzymes. Mutation of the catalytic serine residue within the GXSXG lipase consensus sequence abolished the ability of the enzyme to hydrolyze either phospholipid or lysophospholipid substrates (Figure 1b–c).

### Mutations associated with INAD and NBIA cause loss of enzyme activity

**Figure 2:**
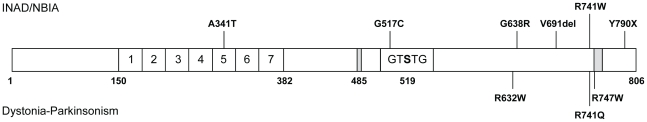
Disease-associated PLA2G6 mutations examined in this study and their relationship to functional domains of the PLA2G6 protein. Illustrated functional domains in the PLA2G6 protein (encoded by transcript variant 1) include the ankyrin repeat regions (numbered regions between amino acids 150–382) and the GXSXG lipase catalytic site (S519). Shaded regions indicate a nucleotide binding domain centered at amino acid 485, and a calmodulin binding region (amino acids 747–759). The locations of amino acid changes resulting from mutations associated with INAD/NBIA are indicated above the diagram and mutations associated with dystonia-parkinsonism are indicated below the diagram.

We used site directed mutagenesis to introduce missense mutations previously identified in patients diagnosed with either NBIA or INAD. These mutations included Y790X, the most frequent mutation found in association with INAD/NBIA that results in a premature stop codon, truncating the last 15 amino acids of the WT protein. We selected several other mutations based on their location within proposed functional regions of PLA2G6, including the ankyrin repeat region that may be responsible for protein-protein interactions (A341T), the first glycine residue in the GXSXG lipase domain (G517C), and a C-terminal region that includes a calmodulin binding domain (G638R). The location of disease-associated mutations relative to functional domains in the PLA2G6 protein is illustrated in Figure 2. We produced recombinant proteins containing each of the disease-associated mutations and compared catalytic activity to the WT PLA2G6 protein. To compare specific activities of WT and mutant proteins, quantitative western blot analysis was used to determine the relative PLA2G6 protein concentration for each recombinant protein preparation and add equal amounts of WT and mutant proteins to the catalytic assays.

**Figure 3:**
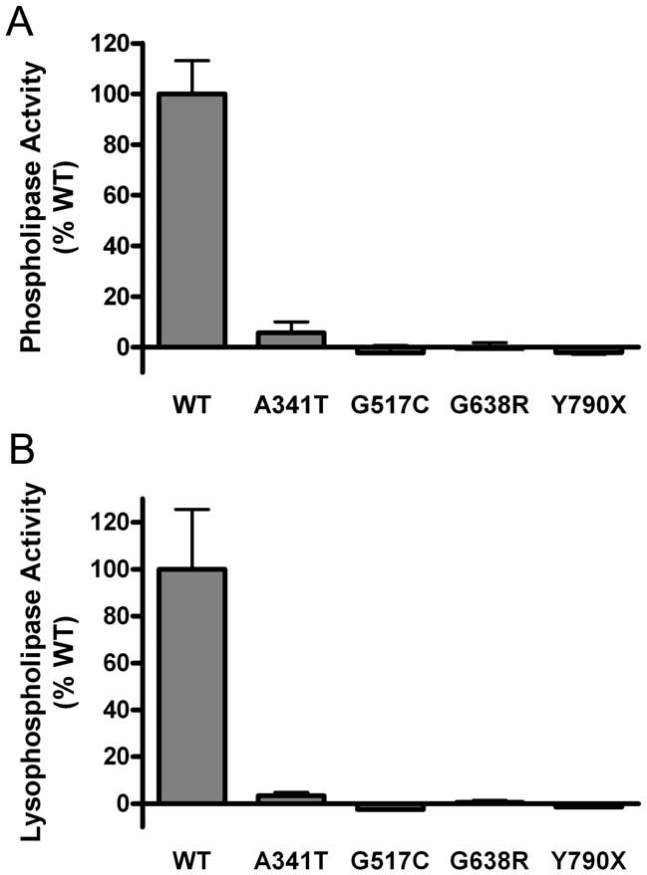
PLA2G6 mutations that cause NBIA/INAD significantly disrupt the ability of PLA2G6 to hydrolyze PC and LPC. Phospholipase and lysophospholipase catalytic activities of mutant PLA2G6 proteins were compared to wild type (WT) PLA2G6 in assays with PC and LPC substrates, respectively. Initial experiments examined the group of INAD-associated mutations A341T, G517C, G638R, and Y790X. Phospholipase (A) and lysophospholipase (B) activities are shown as percent of WT activity measured when equal concentrations of protein were added to assays measuring release of fatty acids from radiolabeled substrate. Bars indicate mean plus standard deviation (n = 3) from representative experiments. The specific activities of all mutations were significantly different from WT (p<0.05, unpaired t-test). Similar results were obtained in at least two experiments with independent transfection and purification of recombinant proteins.

We found that the above mutations associated with INAD/NBIA significantly reduced PLA2G6 phospholipase activity compared to WT protein. The A341T, G517C, G638R, and Y790X mutant proteins had less than 10% of the activity of WT protein (Figure 3A). We also examined the catalytic activity of mutant proteins using the substrate LPC. The lysophospholipase assay yielded results that were similar to those in the phospholipase assay, with INAD-associated mutations causing impaired enzyme activity for the LPC substrate (Figure 3B).

**Figure 4:**
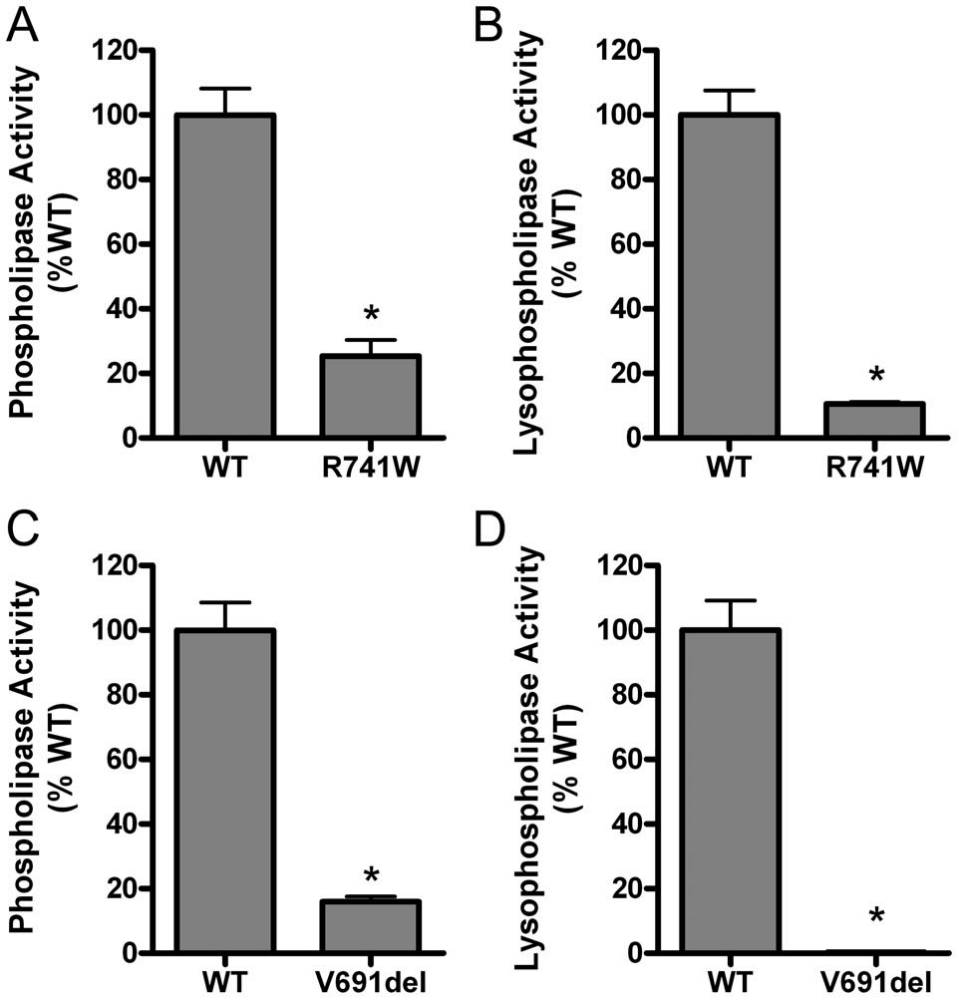
The effects of additional mutations illustrate the significance of genotype distinctions between INAD/NBIA and dystonia-parkinsonism. The INAD/NBIA-associated R741W mutation (A, B) was investigated because mutation of the same residue to a glutamine (R741Q) has been identified in dystonia-parkinsonism. The V691del mutation was examined given its association with an R632W mutation in an INAD patient with compound heterozygous mutations (C, D), in contrast to homozygous R632W mutations associated with dystonia-parkinsonism. Phospholipase (A,C) and lysophospholipase (B,D) activities are shown as percent of WT activity measured when equal concentrations of protein were added to assays measuring release of fatty acids from radiolabeled substrate. Bars indicate mean plus standard deviation (n = 3) from representative experiments. Asterisks indicate mean activities that were significantly different from WT (p<0.05, unpaired t-test). Similar results were obtained in at least two experiments with independent transfection and purification of recombinant proteins.

To further investigate the potential significance of genotype differences between INAD/NBIA and dystonia-parkinsonism, we examined the catalytic activity of two other mutations associated with NBIA/INAD. Mutations causing a missense amino acid substitution at position 741 are associated with both INAD/NBIA (R741W) and dystonia-parkinsonism phenotypes (R741Q). We examined the effect of the INAD/NBIA-associated tryptophan substitution at amino acid 741 (R741W) and found that this amino acid change reduced PLA2G6 phospholipase activity to approximately 25% of the level of WT activity and lysophospholipase activity to approximately 10% of WT activity (Figure 4A–B). We also examined the catalytic activity of the V691del mutation because it has been identified in combination with an R632W mutation in an INAD patient with compound heterozygous mutations, which is in contrast to homozygous R632W mutations associated with dystonia-parkinsonism. To investigate the potential contribution of the V691del mutant allele observed in combination with an R632W allele in INAD/NBIA, we used site directed mutagenesis to delete valine 691. The V691del mutation reduced phospholipase activity to15% of WT, and lysophospholipase activity to less than 1% of WT (Figure 4C–D).

### Mutations that cause dystonia-parkinsonism do not impair PLA2G6 catalytic activity

**Figure 5:**
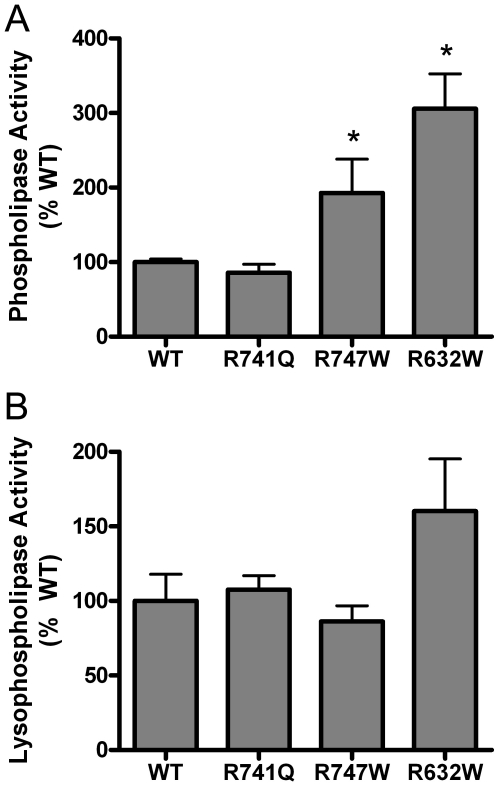
PLA2G6 mutations associated with dystonia-parkinsonism do not impair phospholipase and lysophospholipase activity. Purified recombinant protein was produced for WT PLA2G6 protein and for PLA2G6 proteins containing missense mutations associated with dystonia-parkinsonism, Equal amounts of WT or mutant proteins were added to enzymatic assays utilizing (A) PC or (B) LPC as substrate. Relative rates of fatty acid release are shown for WT and each mutant protein (mean percent WT + standard deviation, n = 3 independently prepared protein preparations for each of WT and 3 mutants). Asterisks indicate mean activities that were significantly different from WT (p<0.05, unpaired t-test). Although an initial experiment with n = 1 recombinant protein preparations indicated decreased activity of all three mutant proteins relative to WT, results similar to those shown in panels A and B were observed in all subsequent experiments, which included at least 3 additional independent protein preparations.

We also produced recombinant PLA2G6 proteins containing the three mutations associated with dystonia-parkinsonism (R632W, R741Q, and R747W). In contrast to the effects of INAD/NBIA-associated mutations, the three mutations associated with dystonia-parkinsonism did not impair phospholipase catalytic activity (Figure 5). In fact, PLA2G6 enzyme with the R747W and R632W mutations displayed an increased rate of PC-hydrolysis relative to WT enzyme in the phospholipase assay. In the lysophospholipase assay, the catalytic rates of the three mutant proteins were not significantly different from WT. Since PLA2G6 has also been observed to hydrolyze palmitoyl coenzyme A (CoA) in vitro [9], we examined PLA2G6 catalytic activity using radiolabeled palmitoyl CoA, and observed that the dystonia-parkinsonism mutations also do not impair PLA2G6 thioesterase activity (Figure S1).

## Discussion

Our results provide insight into pathogenic mechanisms underlying the spectrum of neurodegenerative diseases caused by *PLA2G6* mutations. We find that mutations associated with NBIA/INAD impair the catalytic activity of the PLA2G6 protein. In contrast, mutations associated with dystonia-parkinsonism do not impair catalytic activity. These results clarify the mechanisms underlying the phenotypic expression associated with *PLA2G6* mutations; selective effects on protein function, rather than other genetic or environmental factors, produce the two different disease spectrums, NBIA/INAD and dystonia-parkinsonism. Comparison of the effects of a glutamine (Q) versus a tryptophan (W) substitution at the 741 position further illustrates the correlation between enzymatic activity and disease phenotype. A tryptophan substitution for arginine at position 741 produces an 80% reduction in activity, associated with INAD in one patient and NBIA in another patient, while glutamine substitution results in no apparent reduction in catalytic activity and is associated with a dystonia-parkinsonism phenotype.

Although our studies do not detect a loss of catalytic function resulting from dystonia-parkinsonism mutations, the recessive pattern of inheritance suggests that they are more likely to cause a loss of function rather than a dominant gain of function. The R632W mutation has been observed in one patient with INAD in addition to three siblings with dystonia-parkinsonism. In the patient with INAD, a heterozygous R632W mutation was found in combination with a heterozygous V691del mutation, which is distinct from the situation in dystonia-parkinsonism patients who have all been homozygous for the R632W mutation. The complete loss of enzyme function caused by the V691del mutation suggests that a genotype-phenotype correlation may exist based on the degree of impairment in PLA2G6 function, and that impairment below a certain level may cause the early onset INAD/NBIA disease phenotype and more widespread effects in the nervous system. Although our assays did not detect impairment of catalytic activity, they do not exclude the possibility of impaired enzyme function in vivo. An alternative explanation is that an additional *PLA2G6* mutation was present in this patient but was not detected by sequencing of *PLA2G6* exons.

Our results suggest that the R632W and R747W mutations might alter PLA2G6 function by increasing the catalytic rate for PC. We did not observe an increase in the catalytic rate for LPC. Further experiments are needed to determine whether these mutations alter the relative catalytic rates for PC and LPC. Such a change in substrate preference could significantly alter enzyme function in vivo. By promoting phospholipid hydrolysis over lysophospholipid hydrolysis, the mutations may alter the relative levels of phospholipids, lysophospholipids, and fatty acids normally regulated by the PLA2G6 enzyme. It is also possible that these mutations interfere with other mechanisms regulating PLA2G6 function, such as interactions with calmodulin, calcium/calmodulin-dependent protein kinase IIβ or other proteins [10], [11], which may not be detected in our in vitro assays.

The capacity for human PLA2G6 to hydrolyze both phospholipid and lysophospholipid substrates supports a role for the enzyme in phospholipid homeostasis, which is also supported by histopathological changes in INAD and experimental results in cultured cells. In cultured cell lines, PC levels remain constant in the face of increased PC synthesis produced by over-expression of a rate-limiting PC synthesis enzyme. Cells compensate for increased PC production by the conversion of PC to free fatty acids and glycerophosphocholine (GPC), a process that is associated with increased expression of PLA2G6 and blocked by an inhibitor of PLA2G6 [12], [13]. Our results for human PLA2G6, consistent with previous studies of Chinese hamster PLA2G6 [8], [14], demonstrate the capacity of PLA2G6 to catalyze both enzymatic steps in the conversion of PC to fatty acids and GPC.

The proposed role for PLA2G6 in phospholipid turnover does not exclude additional roles in membrane remodeling or arachidonic acid signaling pathways, but a role in phospholipid homeostasis may explain the axonal accumulation of membranes in neuroaxonal spheroids, the hallmark feature of NBIA/INAD observed throughout the peripheral and central nervous system [15]–[21] The core component of neuroaxonal spheroids is tubulovesicular membrane accumulation, and this feature is reproduced in mouse models of INAD produced by mutations in the *PLA2G6* gene [22]–[24], including a missense mutation that disrupts PLA2G6 phospholipase activity [23]. Interestingly mice with mutations in the PLA2G6 gene develop neurological impairment later within the mouse lifespan than observed in human INAD [22], [23]. Later onset disease in the mouse may result from differences between species in neuronal vulnerability to loss of PLA2G6. Species differences in axon length, metabolic requirements or compensatory metabolic pathways could explain differences in age of onset and degree of neurological impairment.

Our results predict that *PLA2G6* mutations may cause not only accumulation of phospholipid substrates but also decreased production of fatty acids. Fatty acid release by PLA2G6 may be important in the synthesis of new phospholipids, triglycerides and other lipids, or alternatively catabolic pathways such as fatty acid beta-oxidation. The INAD/NBIA phenotype is also associated with accumulation of alpha-synuclein in Lewy bodies and Lewy neurites [4]. Since alpha-synuclein binds fatty acids and regulates brain fatty acid metabolism [25]–[30], altered fatty acid metabolism may be a mechanism underlying alpha-synuclein accumulation. Additional studies in neuronal culture and mouse models may further define mutation-induced changes in PLA2G6 function, and mechanisms linking changes in PLA2G6 catalytic activities to these histopathological features of neurodegeneration.

## Materials and Methods

### Materials

Chemicals were obtained from Sigma unless otherwise indicated.

### Plasmids

The full length human *PLA2G6* coding sequence for transcript variant 1 was amplified by PCR from the human cDNA clone MGC:45156 (IMAGE:5166749, Genbank:BC036742), using forward primer aagaattcaccatgcagttctttggccgcctg and reverse primer aatctagagggtgagagcagcagctggat. The forward primer contains an added EcoRI restriction site upstream of the ATG start codon and the reverse primer contains an XbaI site downstream of the last codon. The PCR product was subcloned into pcDNA3.1 myc-his. Mutations were generated in the *PLA2G6* transcript variant 1 pcDNA 3.1 expression vector using the Quickchange Site-Directed Mutagenesis protocol (Stratagene, La Jolla, CA). Mutations were confirmed by sequencing.

### Production and purification of recombinant PLA2G6 protein

293FT cells (Invitrogen) were cultured in DMEM containing 10% fetal bovine serum (FBS), penicillin and streptomycin. Cells were transfected in 10 cm culture dishes using Lipofectamine 2000, by combining 36 µl of Lipofectamine 2000 reagent and 12 µg plasmid DNA in a total volume of 3 ml Optimem medium. After incubating the mixture for 20 min at room temperature, it was added dropwise to 5 ml DMEM with 10% FBS in a 10 cm tissue culture dish. To this mixture were added 9×10^6^ 293 FT cells suspended in 5 ml DMEM with 10% FBS, which were prepared by trypsin digestion and trituration. The DNA liposome-containing medium was replaced with growth medium the next morning and cells were cultured for an additional day before extraction of protein at approximately 42 hours after transfection (longer incubation times after transfection led to significant cell death that was specific to PLA2G6 plasmids). After washing in 5 ml phosphate buffered saline, cells were extracted in 1.25 ml Triton Wash Buffer (0.1% Triton X-100, 50 mM TrisHCl pH 8.0, 500 mM NaCl, 20 mM imidazole, 2 mM 2-mercaptoethanol, 5 µg/ml aprotinin and 5 µg/ml leupeptin. The extract was frozen at −80°C, thawed, sonicated with five pulses of 1 sec, and centrifuged at 15,000× g for 10 min. The supernatant was combined with 0.1 ml Ni-NTA Agarose beads (Invitrogen) equilibrated in Triton wash buffer, and incubated with gentle rocking for 30 min at 4°C. Beads were collected from the suspension using a 0.8 ml Handee spin column (Pierce) with centrifugation at 500× g. Beads were washed two times in Triton Wash buffer, then washed an additional two times in glycerol wash buffer (same composition as Triton wash buffer except 20% glycerol was substituted for 0.1% Triton X-100). Bound protein was eluted in glycerol elution buffer (20% glycerol, 50 mM TrisHCl pH 8.0, 500 mM NaCl, 250 mM imidazole, 2 mM 2-mercaptoethanol, 5 µg/ml aprotinin and 5 µg/ml leupeptin) using three separate 0.1 ml additions of elution buffer and collection of eluate by centrifugation.

### Quantitation of PLA2G6 protein levels in purified fractions

PLA2G6 protein concentrations in catalytic assays for WT and mutant PLA2G6 were normalized based on quantitative Western blot analysis of purified fractions. Typically 1.2 µl of each purified fraction was loaded on a 26-well Criterion 10% SDS-PAGE gel (Bio-Rad), with 2–3 replicate lanes per protein sample. Electrophoresed proteins were transferred to nitrocellulose membrane, incubated with mouse anti-myc monoclonal antibody 9E10, followed by horseradish peroxidase conjugated anti-mouse secondary antibody (Jackson Immunoresearch). Bound secondary antibody was detected by enhanced chemiluminescence (ECL) using Immobilon Western ECL Substrate (Millipore). Blots were imaged with a Kodak Image Station 4400 and the intensity of individual bands compared using Kodak 1D analysis software. Standard curves containing a range of WT protein amounts were included in the analysis of each set of recombinant proteins, in order to verify that ECL signal for the amount of protein loaded was in the linear range for quantitation. A graph demonstrating the linear relationship between PLA2G6 protein concentration and ECL signal intensity is shown in Figure S2. Average ECL signal intensity was used to determine the relative PLA2G6 protein concentrations in each preparation, which was used to add equal amounts of WT and mutant proteins to the catalytic assays.

### Assays for PLA2G6 catalytic activity

Catalytic activities of purified recombinant enzymes were measured using in vitro reactions containing 25 mM Tris HCl pH 7.5, 1 mM EGTA, and 4.5 µM lipid substrate, using methods similar to previously reported catalytic assays [7]–[9], [31]. Substrates were L-α-1-palmitoyl-2-oleyl phosphatidylcholine [oleyl-1-^14^C] (POPC, from American Radiolabeled Chemicals, 55 mCi/mmol), and Lysopalmitoyl-phosphatidylcholine L-1-[palmitoyl-1-^14^C] (LPC, from Perkin Elmer, 55 mCi/mmol), which were supplied in 1∶1 toluene:ethanol. Palmitoyl Coenzyme A [palmitoyl 1-^14^C] (Palmitoyl CoA, from American Radiolabeled Chemicals, 55 mCi/mmol) was supplied in 1∶1 0.01M sodium acetate:ethanol. Prior to experiments, the substrate was evaporated under a stream of nitrogen and redissolved in 100% ethanol at a concentration of 100 µM. The aqueous portion of the reaction, minus the enzyme volume, was rapidly mixed with the ethanol-dissolved substrate, and the reaction mixture was then sonicated in either a cup horn or bath sonicator for 20 minutes at 25°C prior to addition of enzyme. Immediately after addition of enzyme in a 5 µl volume, reactions (50 µl final volumes) were incubated at 37°C for 2 min for phospholipase assays or 4 min for lysophospholipase assays. Fatty acid products were extracted by addition of butanol (25 µl) immediately after the incubation period, followed by vortexing and centrifugation at 2000× g for 4 minutes. Butanol-extracted lipids were separated by thin layer chromatography on silica gel plates (Partisil LK6F, Whatmann) using a 80∶20∶1 petroleum ether:diethyl ether:acetic acid solvent system. TLC plates were exposed to image phosphor plates, which were analyzed using a FLA-7000 phosphorimager and Multigauge software (Fujifilm). Control reactions were included in each experiment and contained equivalent volumes of purified protein from vector-transfected cells. The average amount of product obtained in control reactions (typically less than 5% of product produced by WT PLA2G6 preparations) was subtracted to obtain the final value for each enzyme preparation. Less than 5% of substrate was converted to product under the reaction conditions used in experiments, and for the amount of enzymes added to the reactions, product formation was linear with respect to enzyme concentration (Figure S3) and was also linear with respect to incubation time (not shown).

## Supporting Information

Figure S1 PLA2G6 mutations associated with dystonia-parkinsonism do not impair PLA2G6 thioesterase activity. WT or mutant proteins were added at equal enzyme concentrations to catalytic assays utilizing radiolabeled palmitoyl CoA as substrate. Relative rates of fatty acid release are shown for WT and each mutant protein (mean percent WT + standard deviation, n = 3 independently prepared protein preparations for each of WT and 3 mutants). The asterisk indicates that the mean activity of R632W was significantly different from WT (p<0.05, unpaired t-test). Similar to the results observed in phospholipase and lysophospholipase assays, mutations associated with dystonia-parkinsonism do not impair the thioesterase catalytic activity of PLA2G6.
(0.17 MB TIF)

Figure S2 Western blot analysis of purified recombinant protein to normalize WT and mutant protein concentrations in catalytic assays. The graph shows the western blot ECL signal measured for different amounts of purified WT PLA2G6 protein (diluted 15, n = 3 lanes for each volume). The graph also shows a standard curve (y = 16400x-1184) obtained by linear regression (R2 = 0.92) for the relationship between luminescence (after subtracting average luminescence of control lanes from vector transfected cells) and volume of WT PLA2G6 protein. Western blot analysis was used to determine the relative PLA2G6 protein concentration in each recombinant protein preparation and to normalize the amount of protein added to the catalytic assay as outlined in Material and Methods.
(0.26 MB TIF)

Figure S3 Linear relationship between enzyme concentration and free fatty acid production in catalytic assays used to compare specific activities of WT and mutant PLA2G6 enzymes. Different amounts of WT enzyme were added to a catalytic assay with ^14^C-labeled LPC. Released free fatty acids were separated on TLC and quantified by phosphorimager. The graph shows a linear relationship between enzyme concentration and fatty acid release. In experiments examining the effects of mutations associated with dystonia-parkinsonism, the amount of enzyme added to the assays was equivalent to 2 µl WT PLA2G6 enzyme in the above experiment.
(0.19 MB TIF)
