# Supplementary figures and images for: Catalytic Function of PLA2G6 Is Impaired by Mutations Associated with Infantile Neuroaxonal Dystrophy but Not Dystonia-Parkinsonism
Source: PLoS One. 2010 Sep 23;5(9):e12897. doi: 10.1371/journal.pone.0012897 (PMC2944820; doi:10.1371/journal.pone.0012897)

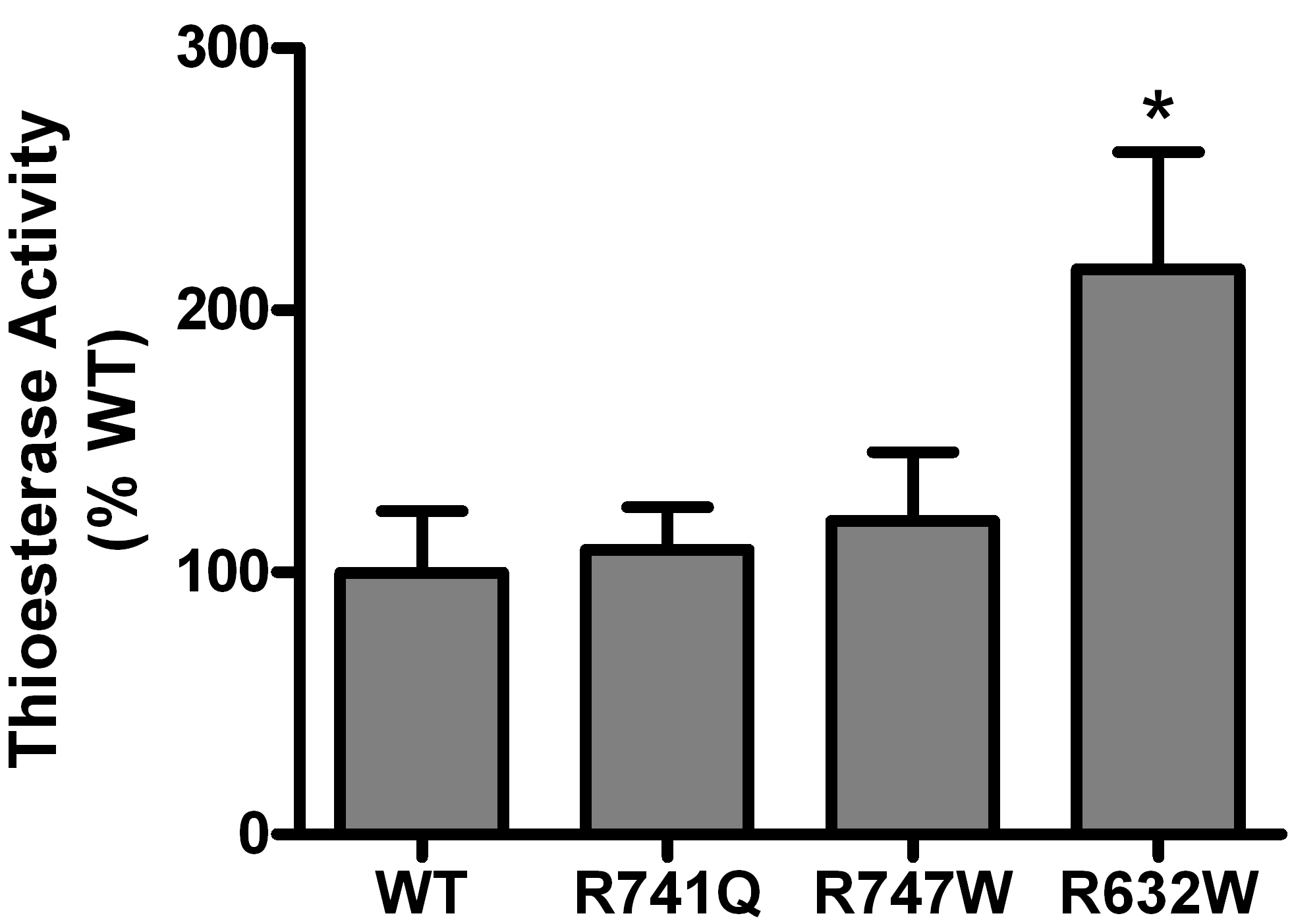

Supplement: Figure S1 — PLA2G6 mutations associated with dystonia-parkinsonism do not impair PLA2G6 thioesterase activity. WT or mutant proteins were added at equal enzyme concentrations to catalytic assays utilizing radiolabeled palmitoyl CoA as substrate. Relative rates of fatty acid release are shown for WT and each mutant protein (mean percent WT + standard deviation, n = 3 independently prepared protein preparations for each of WT and 3 mutants). The asterisk indicates that the mean activity of R632W was significantly different from WT (p<0.05, unpaired t-test). Similar to the results observed in phospholipase and lysophospholipase assays, mutations associated with dystonia-parkinsonism do not impair the thioesterase catalytic activity of PLA2G6. (0.17 MB TIF) [file pone.0012897.s001.tif]

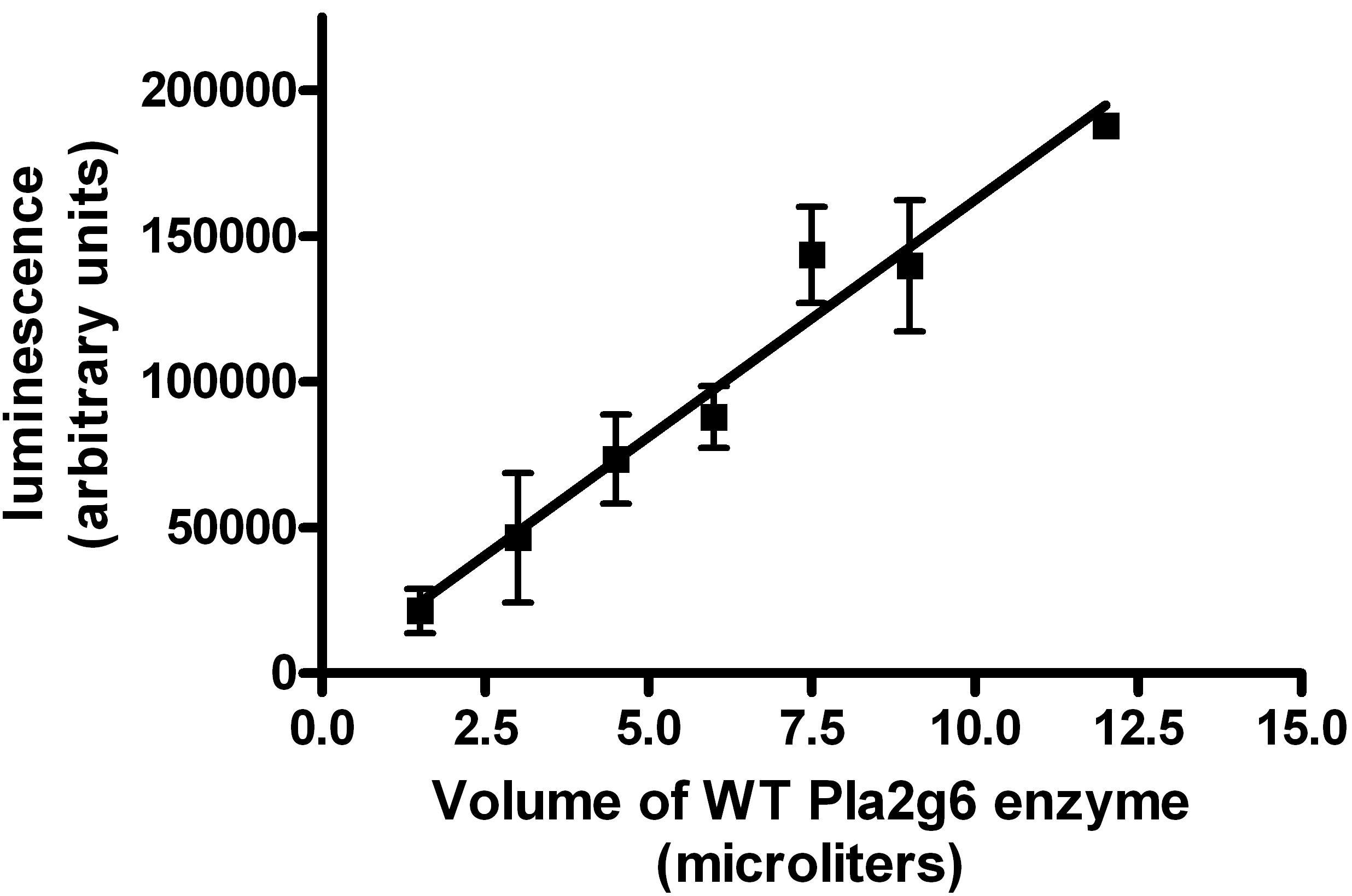

Supplement: Figure S2 — Western blot analysis of purified recombinant protein to normalize WT and mutant protein concentrations in catalytic assays. The graph shows the western blot ECL signal measured for different amounts of purified WT PLA2G6 protein (diluted 15, n = 3 lanes for each volume). The graph also shows a standard curve (y = 16400x-1184) obtained by linear regression (R2 = 0.92) for the relationship between luminescence (after subtracting average luminescence of control lanes from vector transfected cells) and volume of WT PLA2G6 protein. Western blot analysis was used to determine the relative PLA2G6 protein concentration in each recombinant protein preparation and to normalize the amount of protein added to the catalytic assay as outlined in Material and Methods. (0.26 MB TIF) [file pone.0012897.s002.tif]

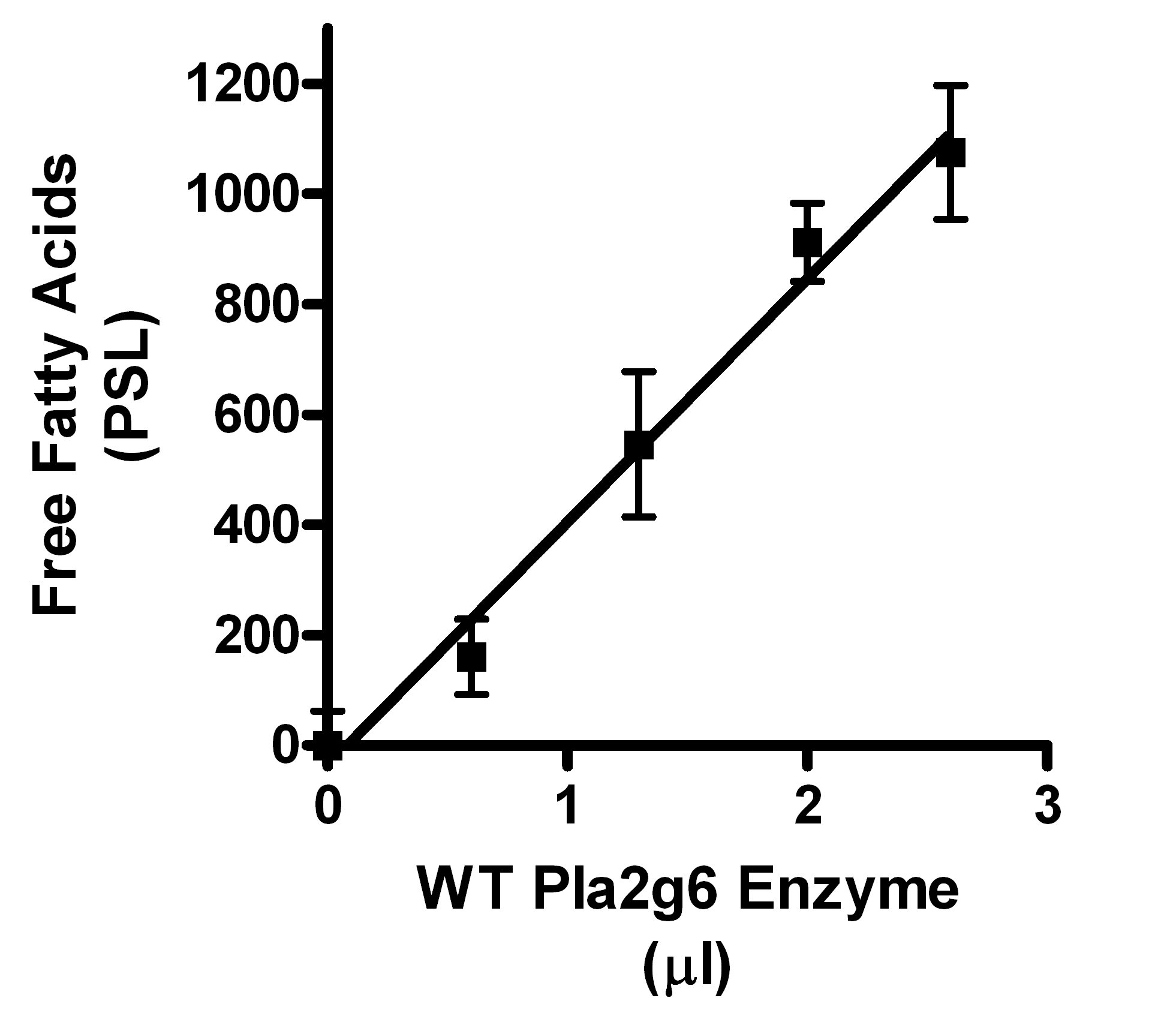

Supplement: Figure S3 — Linear relationship between enzyme concentration and free fatty acid production in catalytic assays used to compare specific activities of WT and mutant PLA2G6 enzymes. Different amounts of WT enzyme were added to a catalytic assay with 14C-labeled LPC. Released free fatty acids were separated on TLC and quantified by phosphorimager. The graph shows a linear relationship between enzyme concentration and fatty acid release. In experiments examining the effects of mutations associated with dystonia-parkinsonism, the amount of enzyme added to the assays was equivalent to 2 µl WT PLA2G6 enzyme in the above experiment. (0.19 MB TIF) [file pone.0012897.s003.tif]
